# Are individual differences in personality associated with COVID-19 infection? Examining the role of normative, maladaptive, and dark personality traits using structural equation modeling

**DOI:** 10.3389/fpsyg.2025.1511970

**Published:** 2025-02-19

**Authors:** Parandis Pourdehghan, Aaron L. Pincus, Mohammad Reza Mohammadi

**Affiliations:** ^1^Department of Psychology, Pennsylvania State University, University Park, PA, United States; ^2^Psychiatry and Psychology Research Center, Roozbeh Hospital, Tehran University of Medical Sciences, Tehran, Iran

**Keywords:** personality, personality pathology, individual differences, social distancing, COVID-19

## Abstract

**Objective:**

During the COVID-19 pandemic, people’s behaviors have been considered an important factor in the spread of coronavirus. This situation led us to examine the role of personality in human behavior and its outcomes during the pandemic. This study examined the effect of normative, maladaptive, and dark personality traits on the probability of COVID-19 infection as mediated by psychological and behavioral responses to the pandemic.

**Methods:**

The data was collected from 740 Iranians (mean age = 33.34) completing Big Five-10, Personality Inventory for DSM-5-Brief Form (PID-5-BF)-Adult, Short Dark Triad (SD3), Depression, Anxiety and Stress Scale - 21 Items (DASS-21), and Protective Behaviors inventories. We used structural equation modeling to fit a model from the personality traits to COVID-19 infection through mediating effects of psychological and behavioral responses using cross-sectional data.

**Results:**

All path models examined fit the data well. The normative traits openness, conscientiousness, neuroticism, introversion, and disagreeableness were positively related to social distancing. The pathological traits antagonism, detachment, negative affectivity, disinhibition, and psychoticism, and dark traits psychopathy, narcissism, and Machiavellianism were negatively associated with social distancing. Finally, social distancing was negatively related to infection rates and fully mediated all personality links with infection (*β* = −0.17, *p* < 0.001).

**Conclusion:**

The findings demonstrate that individual differences in personality predict behaviors crucial to pandemic mitigation. Social distancing can be, directly or indirectly, a significant underlying mechanism linking personality traits to the COVID-19 infection. Public health policymakers should consider personality-tailored interventions for maximizing preventive health behaviors and slowing the spread of infection. This knowledge also could contribute to more effective measures to prepare for public health emergencies in the future.

## Introduction

The Coronavirus Disease 2019 (COVID-19) is an unprecedented pandemic that quickly spread worldwide, with 774 million confirmed cases and more than 7 million deaths universally as of January 2024 according to the World Health Organization (WHO). The world has struggled with the COVID-19 pandemic spreading from person to person. One important issue for controlling the outbreak is how people respond and behave during the pandemic (Anderson et al., 2020; Gotz et al., 2021). For instance, engaging in protective behaviors is crucial to reduce the spread of the virus, however, there are individual differences in how vigilantly people follow such behaviors (Aschwanden et al., 2020). An important goal for research is to determine what factors lead to variability in following recommended protective behaviors.

Personality is generally regarded as a key factor in health-related behaviors, risk, outcomes, and even interventions (Friedman and Kern, 2014; Juchem et al., 2024; Williams and Carlson, 2025). Based on theory and research in the psychological and social sciences, we can consider the role of personality traits as psychological determinants of the social and compliance behaviors that drive or mitigate the spread of COVID-19 infection (Betsch, 2020; Block et al., 2020). Some studies demonstrate that individual differences in personality are associated with behavior (e.g., social distancing, sheltering in place) even when governments take intensive action targeting that behavior (Gotz et al., 2021). Individual differences in general patterns of thought, feeling, and behaviors (personality traits) predict specific behaviors and social outcomes (Ozer and Benet-Martinez, 2006; Roberts et al., 2007), including behaviors related to the COVID-19, such as psychological responses, social distancing, and other compliance behaviors (Aschwanden et al., 2020; Blagov, 2021).

Personality Traits and COVID-19 Pandemic Responses.

Although general studies showing how personality traits are associated with health behaviors are useful, examining this in a special situation, the COVID-19 pandemic, can give us a clearer vision of the role of personality traits in a critical situation. Because the COVID-19 pandemic is a global challenge, international research across countries and cultures is vital (Absetz et al., 2025; Tiwari et al., 2024). Previous research across several countries suggests multiple reasonable links between Big Five personality traits and behavioral and psychological responses to the COVID-19 pandemic that might be related to protective behaviors in different ways (Gotz et al., 2021). A study in the US and Germany showed that social distancing and compliance behaviors were positively associated with the level of Openness, Conscientiousness, Agreeableness, and Neuroticism, and negatively related to Extraversion (Peters et al., 2020). A study in the US (Aschwanden et al., 2020) also showed how personality predicts concerns and behaviors related to the COVID-19 pandemic; for example, higher Conscientiousness was associated with more precautions, and higher Neuroticism was associated with fewer precautions. A study in South Korea found that Agreeableness and Conscientiousness had a positive association with preventive behaviors (Han et al., 2021). Some studies have also examined the association between normative personality traits and psychological responses during the pandemic. For instance, a German study found that individuals high in Neuroticism experienced more negative affect in their daily lives during the pandemic, and the impact of Neuroticism on negative affect was far greater than that of sociodemographic variables and experienced health threats (Kroencke et al., 2020).

Maladaptive (antagonism, detachment, negative affectivity, disinhibition, psychoticism) and dark triad (narcissism, psychopathy, Machiavellianism) personality traits have received much less attention than normal personality traits during the COVID-19 pandemic. Regarding maladaptive personality traits, one study found that disinhibition predicted a lower predisposition to engage in social distancing and hygiene protective behaviors (Blagov, 2021). Previous research across several countries also showed that negative affectivity, disinhibition, and detachment were positively associated with depression, anxiety, and stress during the pandemic (Han et al., 2021; Sica et al., 2021a; Somma et al., 2020). Persons characterized by dark personality traits were also less likely to follow protective restrictions related to COVID-19 (Zajenkowski et al., 2020). Blagov (2021) found that dark personality traits were negatively associated with social distancing and hygiene-related health behaviors during the COVID-19 pandemic in an American sample. German research showed that dark personality traits were negatively related to accepting personal restrictions to fight COVID-19 (Modersitzki et al., 2021). Further, research conducted during the pandemic showed that psychopathy was associated with high stress and negative affect (Sica et al., 2021b). The results of a study in Poland showed a significant positive correlation between the Dark Triad and depressive symptoms, alongside the link between narcissism and anxiety symptoms (Gogola et al., 2021). Additionally, a few studies (Peters et al., 2023; Rolon et al., 2021) have examined links between personality traits, health behaviors, and COVID-19 infection itself simultaneously.

To further the global investigation of personality traits and COVID-19 pandemic responses, the current cross-sectional study is the first to examine associations between personality traits and both COVID-19 infection and pandemic related behavioral and psychological responses in a large sample of Iranian adults. Additionally, this study extends prior research by examining whether the associations between personality traits and infection are mediated by the pandemic-related psychological and behavioral responses. Such processes might have implications for health services and public health officials’ understanding how personality has a role in the COVID-19 pandemic as it can be useful to anticipate people’s behaviors during infectious disease pandemics and provide personality-based advice for public health services.

## Method

### Participants

We collected data online in the Persian language between early November 2021 and late January 2022, a time period coinciding with the peak of the COVID-19 pandemic in Iran. During this time frame, the government enforced regulations related to the pandemic, such as social distancing, compliance with hygiene rules, and vaccination. Individuals were invited to participate using a variety of sources, including social media platforms and social networks. Exclusion criteria included age under 18 years, intellectual disability, and residing abroad. During the time period, 2,194 people received the survey and 800 fully completed surveys were returned. Of these, 54 surveys were removed because the participant was under 18 years old and 6 surveys were removed because the participant resided outside Iran. The final sample included 740 adult participants aged 18–74 years [72% female; mean age = 33.34 years (SD = 11.31)]. Participants provided basic sociodemographic information and completed measures of personality traits, psychological responses, and behavioral responses, and reported if they had experienced a confirmed COVID-19 infection based on positive polymerase chain reaction (PCR) test results or a physician’s diagnosis. COVID-19 infection was assessed as a dichotomous variable- Yes or No.

### Measures

#### Big Five Inventory-10

We used BFI-10 because it allows for a quick and efficient evaluation of the normative personality traits, making it ideal for situations where participants have limited time to complete the survey. Normative personality traits were assessed with the Persian translation of the Big Five Inventory-10 [BFI-10; Mohammad Zadeh and Najafi (2010) and Rammstedt and John (2007)], a 10-item inventory measuring Openness to experience, Conscientiousness, Extraversion, Agreeableness, and Neuroticism based on the 44-item Big Five Inventory (John et al., 1991). BFI-10 items are rated on a five-point Likert scale. Cronbach’s alpha reliability of Extraversion, Agreeableness, Conscientiousness, Neuroticism, Openness in current study were 0.10, 0.10, 0.48, 0.56, 0.18, respectively. These can be interpreted as “average inter-item correlations” which can be evaluated as acceptable for broader constructs (0.10–0.20), moderately broad constructs (0.20–0.40), and narrower constructs (0.40–0.50). We had broader constructs in our study (Clark and Watson, 2019).

#### Personality Inventory for DSM-5-Brief Form (PID-5-BF)-Adult

Maladaptive personality traits were assessed with the Persian translation of the PID-5-BF-Adult (Abdi and Chalabianlou, 2017; Krueger et al., 2013), a 25-item inventory measuring the trait domains of negative affectivity, detachment, antagonism, disinhibition, and psychoticism. Items are rated on a 4-point Likert scale. Cronbach’s alpha reliability of Negative Affectivity, Detachment, Antagonism, Disinhibition, Psychoticism in current study was 0.74, 0.71, 0.60, 0.71, 0.73, respectively.

#### Short Dark Triad (SD3)

Dark Triad traits with the Persian translation of the SD3 (Atari and Chegeni, 2016; Jones and Paulhus, 2014), a 27-item inventory measuring Machiavellianism, psychopathy, and narcissism. Items are rated on a 5-point Likert scale. Cronbach’s alpha reliability of Machiavellianism, Narcissism, Psychopathy in current study was 0.77, 0.67, 0.66, respectively.

#### Depression, Anxiety and Stress Scale - 21 Items (DASS-21)

Psychological responses were assessed with the Persian translation of the DASS-21 (Lovibond and Lovibond, 1995; Sahebi et al., 2005), a 21-item inventory measuring depression, anxiety, and stress. It is based on a dimensional rather than a categorical conception of psychological disorder. Cronbach’s alpha reliability of Depression, Anxiety, Stress in current study was 0.93, 0.87, 0.89, respectively.

#### Protective behaviors questionnaire

Behavioral responses were identified using policy statements that assess the extent to which individuals adopt the Iranian government’s transmission mitigation behavioral guidelines including protective behavioral responses. We developed a 10-item questionnaire to assess COVID-19 protective behavioral responses including social distancing (quarantine, limiting travel, staying at home, avoiding crowded areas, using no-contact greetings, physically distancing from others), and compliance with preventive hygiene actions (wearing masks, vaccination, avoiding touching face with unwashed hands, frequently washing hands). A pilot study (*N* = 40) evaluated the content validity of the questions. Cronbach’s alpha reliability of the questionnaire was 0.80. Participants were asked to indicate the extent to which they had engaged in COVID-19 protective behaviors during the past month on a scale from 1 to 5.

### Data analysis

Hypotheses were tested with path modeling using Amos version 24.0 structural equation modeling (SEM) software package. The nonparametric asymptotically distribution-free (ADF) method was utilized to investigate model paths (Hancock and Mueller, 2013). Because of the sensitivity of the chi-square test to large samples, we followed convention and relied on multiple alternative fit indices to evaluate model fit (Hu and Bentler, 1999). The goodness of fit corresponds with chi-square fit statistics/degree of freedom (CMIN/DF) with values <5.0, goodness of fit index (GFI) with values >0.90, adjusted goodness of fit index (AGFI) with values >0.90, the comparative fit index (CFI) with values >0.90, incremental fit index (IFI) with values >0.90, and the root mean square error of approximation (RMSEA) with values <0.08 (Barrett, 2007). For all models, collinearity was examined by the variance inflation factor-VIF- (Vittinghoff et al., 2012) and by bivariate correlations. Heterogeneity in demographic characteristics including sex, history of medical disease and mental disorder, and COVID-19 vaccination was examined using independent samples t-tests.

### Ethics statement

All procedures followed were in accordance with the ethical standards of the Helsinki Declaration. The current project received ethical approval from the research ethics boards of school of medicine-Tehran University of Medical Sciences (Approval ID: IR.TUMS.MEDICINE.REC.1400.306). Informed consent was obtained from all participants.

## Results

Of the 740 participants, 533 (72%) were female and 466 (63%) were married. The participants were aged 18–74 years (mean age: 33.34; SD: 11.31). Table 1 shows participant characteristics. The chi-square test was employed to assess the homogeneity of the distribution of individuals across categorical variables. The findings indicated that the distribution of individuals across gender, marital status, education, income status, medical and mental health history, and COVID-19 vaccination status varied. Thus the prototypical participant in our sample tended to be vaccinated, female, married, have a bachelor’s degree or less, low to moderate income, unemployed, with no history of severe mental or medical illness. However, the prevalence of persons with COVID-19 was not statistically significant at a threshold of less than 0.05, implying that the distribution of this variable was homogeneous ( $x^{2}=3.11,P=0.07$ ).

**Table 1:** Sample characteristics and COVID-19 infection rate (*N* = 740).

Variables	Mean/*N*	%/S.D	*χ* ^2^
Age (years)	33.34	11.31	
Sex			143.61**
Female	533	72	
Male	207	28	
*Marital status*			49.81**
Single	274	37	
Married	466	63	
*Education*			171.83**
High school diploma or less	281	38	
Bachelor’s degree	286	38.6	
Master’s degree or higher	173	23.4	
*Income*			344.67**
Low	403	54.5	
Moderate	324	43.7	
High	13	1.8	
Occupation			28.80**
Unemployed or unpaid	443	59.9	
Employed	297	40.1	
number of family members	3.46	1.36	
History of medical disease			800.04**
No	511	69.1	
Yes	229	30.9	
History of mental disorder			107.46**
No	588	79.5	
Yes	152	20.5	
COVID-19 vaccination			382.46**
No	104	14.1	
Yes	636	85.9	
COVID-19 infection			3.11
No	346	46.8	
Yes	394	53.2	

**p < 0.05.

We constructed the structural equation models and path analyses to determine how personality variables were associated with psychological and behavioral responses and COVID-19 infection. We sought to test a model in which psychological and behavioral variables (stress, depression, anxiety, compliance with hygiene rules, and social distancing) mediated the relationship between personality traits and COVID-19 infection. We examined total direct and indirect effect models linking personality traits and COVID-19 infection by mediating psychological and behavioral responses for each of the three sets of normative, maladaptive, and Dark Triad personality traits. Although all possible paths were tested in the 3 models (see the supplemental materials, Supplementary Tables S1–S3), only the significant paths are depicted in the figures for clarity.

The collinearity ranges examined by VIF were acceptable for all models (Vittinghoff et al., 2012): Model 1 (1.08–5.17), Model 2 (1.41–5.25), Model 3 (1.41–5.25), Model 4 (1.12–5.07), and Model 5 (1.12–5.07). All variables had acceptable kurtosis values ranging from 3 to −3 exhibited acceptable univariate and multivariate normality (Westfall and Henning, 2013). Pearson’s *r* correlations are also presented in the Supplementary Tables S4–S6. Finally, comparison of variables across sex, history of mental disorder, history of medical disease, and COVID-19 vaccination status was examined by independent samples t-tests (Supplementary Tables S7–S10).

### Normative personality traits, psychological, and behavioral responses, and COVID-19 infection

**Figure 1:**
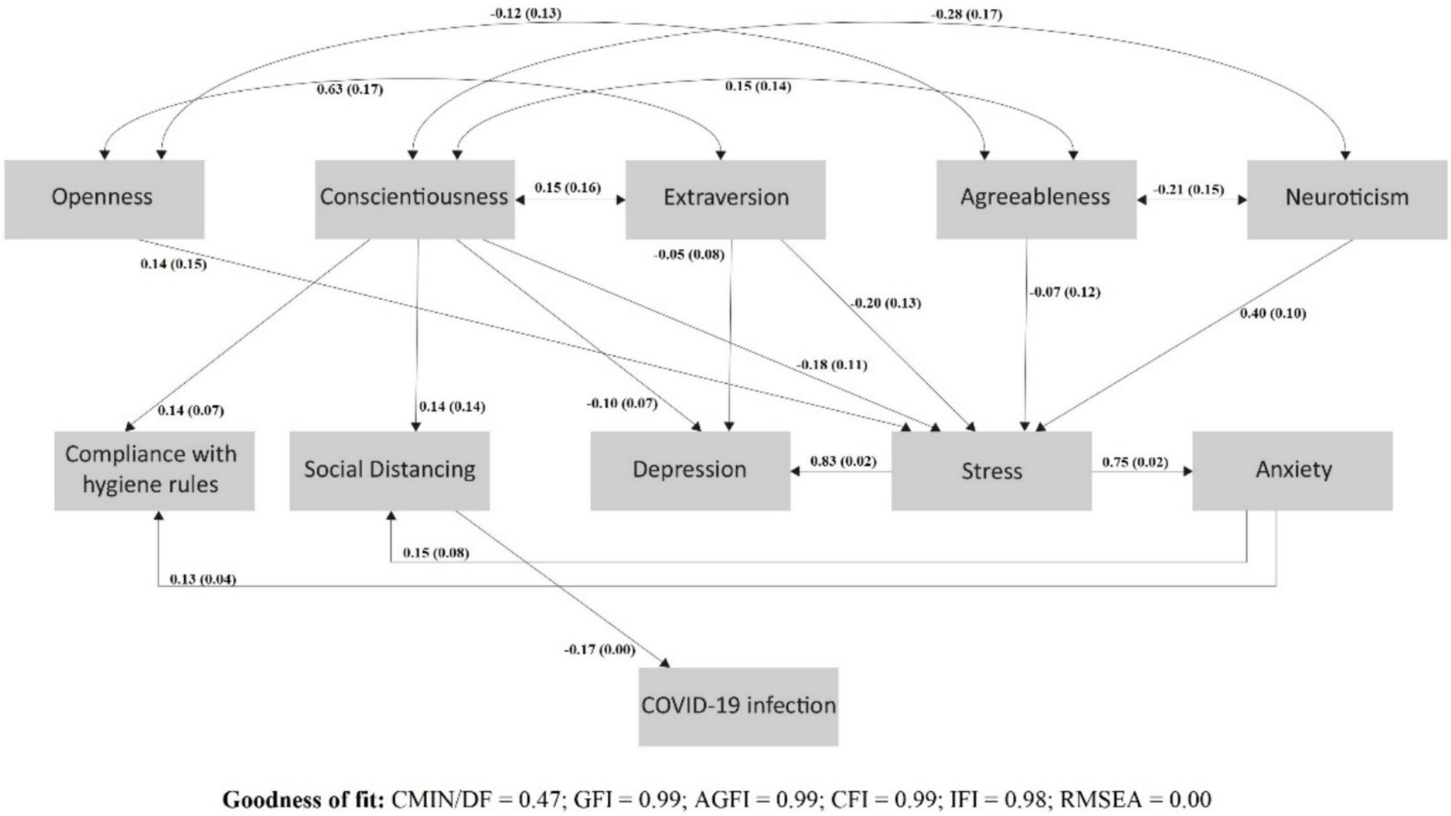
SEM featuring the significant direct and indirect effects of normative personality traits on the COVID-19 infection by mediating social distancing; *β* (S.E).

We hypothesized that normative personality traits would be associated with COVID-19 infection and that these relationships would be mediated by psychological and behavioral responses to the pandemic situation. We tested all possible paths and associations (see supplemental Table S1). Fit indices suggested good model fit [Chi-square = 2.34; Degrees of Freedom (DF) = 5; CMIN/DF = 0.47; GFI = 0.99; AGFI = 0.99; CFI = 0.99; IFI = 0.98; RMSEA ~0.00]. Consistent with our hypothesis, all normative personality traits were significantly associated with COVID-19 infection via social distancing (SD) in the expected directions. Significant paths are presented in Figure 1. Social distancing (SD) was negatively associated with COVID-19 infection. There was a direct significant positive association between conscientiousness and SD. Openness and neuroticism had indirect positive associations with SD (through stress and anxiety), while extraversion and agreeableness had indirect negative associations with SD (through stress and anxiety).

In detail, conscientiousness was directly and positively associated with SD (*β* = 0.144; SE = 0.139; *p* < 0.001), and SD was negatively associated with COVID-19 infection (*β* = −0.167; SE = 0.003; *p* < 0.001). Openness was not associated directly with SD (*β* = −0.004; SE = 0.177; *p* = 0.930), but it was indirectly linked to it via a positive association with stress (*β* = 0.137; SE = 0.145; *p* < 0.001), and stress, in turn, was associated with anxiety (*β* = 0.754; SE = 0.022; *p* < 0.001); anxiety was associated with SD (*β* = 0.146; SE = 0.078; *p* = 0.013), that itself was negatively associated with the infection. Neuroticism also was not associated directly with SD (*β* = −0.021; SE = 0.138; *p* = 0.609), but it was indirectly linked to it via a positive association with stress (*β* = 0.404; SE = 0.104; *p* < 0.001), and then followed the same paths of stress, anxiety, SD, and infection. Extraversion was not associated directly with SD (*β* = −0.015; SE = 0.166; *p* = 0.752), but it was indirectly linked to it via a negative association with stress (*β* = −0.205; SE = 0.134; *p* < 0.001), and then followed the same paths of stress, anxiety, SD, and infection. Agreeableness also was not associated directly with SD (*β* = −0.007; SE = 0.150; *p* = 0.842), but it was indirectly linked to it via a negative association with stress (*β* = −0.066; SE = 0.124; *p* = 0.041), and then followed the same paths of stress, anxiety, SD, and infection. Finally, conscientiousness was directly and positively associated with compliance with hygiene rules (CHR; *β* = 0.138; SE = 0.072; *p* < 0.001). All other normal traits were indirectly associated with CHR via stress, which in turn was positively associated with anxiety (*β* = 0.754; SE = 0.022; *p* < 0.001). Anxiety was positively linked to CHR (*β* = 0.127; SE = 0.040; *p* = 0.029).

### Maladaptive personality traits, psychological and behavioral responses, and COVID-19 infection

**Figure 2:**
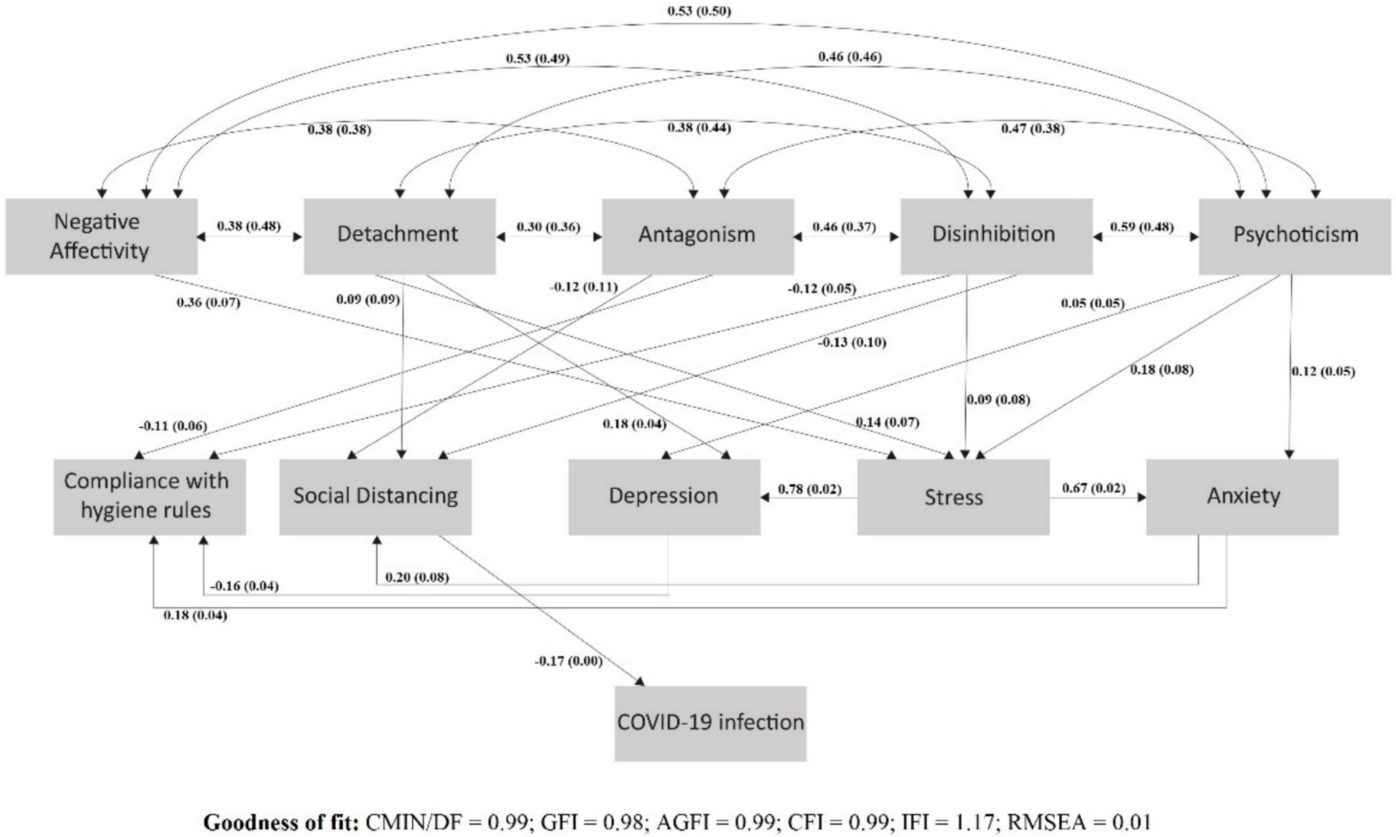
SEM featuring the significant direct and indirect effects of maladaptive personality traits on the COVID-19 infection by mediating social distancing; *β* (S.E).

Figure 2 presents the significant effects of maladaptive personality traits on COVID-19 infection via social distancing. We tested all the paths and associations (see Supplementary Table S2). Fit indices suggested good fit [Chi-square = 7.04; Degrees of Freedom (DF) = 6; CMIN/DF = 1.17; GFI = 0.98; AGFI = 0.99; CFI = 0.99; IFI = 1.17; RMSEA = 0.01]. SD was negatively associated with COVID-19 infection. Detachment was directly positively associated with SD, which in turn had a significantly negative association with COVID-19 infection. In contrast, antagonism and disinhibition were directly and negatively associated with SD, which was negatively associated with infection. Additionally, negative affectivity and psychoticism had indirect positive associations with SD (through stress and anxiety).

In detail, detachment was directly positively associated with SD (*β* = 0.087; SE = 0.093; *p* = 0.047), and SD was negatively associated with the infection (*β* = −0.167; SE = 0.003; *p* < 0.001). Antagonism was directly negatively associated with SD (*β* = −0.115; SE = 0.110; *p* = 0.006), which in turn was negatively associated with the infection. Disinhibition was directly and negatively associated with SD (*β* = −0.127; SE = 0.103; *p* = 0.008), which itself was negatively associated with the infection. Negative affectivity was not associated directly with SD (*β* = −0.015; SE = 0.096; *p* = 0.759), but it was indirectly linked to it via a positive association with stress (*β* = 0.358; SE = 0.070; *p* < 0.001), and stress, in turn, was associated with anxiety (*β* = 0.666; SE = 0.023; *p* < 0.001); anxiety was positively associated with SD (*β* = 0.196; SE = 0.078; *p* < 0.001), which itself had a negative association with the infection (*β* = −0.167; SE = 0.003; *p* < 0.001). Psychoticism also was not associated directly with SD (*β* = 0.015; SE = 0.088; *p* = 0.718), but it was indirectly linked to it via a positive association with stress (*β* = 0.175; SE = 0.081; *p* < 0.001), and then followed the same paths of stress, anxiety, SD, and infection. Finally, Antagonism (*β* = −0.107; SE = 0.056; *p* = 0.010) and Disinhibition (*β* = −0.115; SE = 0.052; *p* = 0.013) were directly and negatively associated with CHR. Other maladaptive traits were indirectly linked with CHR via stress, and stress in turn was positively associated with anxiety (*β* = 0.666; SE = 0.023; *p* < 0.001) and depression (*β* = 0.777; SE = 0.022; *p* < 0.001). Anxiety was positively associated with CHR (*β* = 0.182; SE = 0.040; *p* = 0.002), while depression was negatively associated with CHR (*β* = −0.158; SE = 0.042; *p* = 0.050).

### Dark triad traits, psychological and behavioral responses, and COVID-19 infection

**Figure 3:**
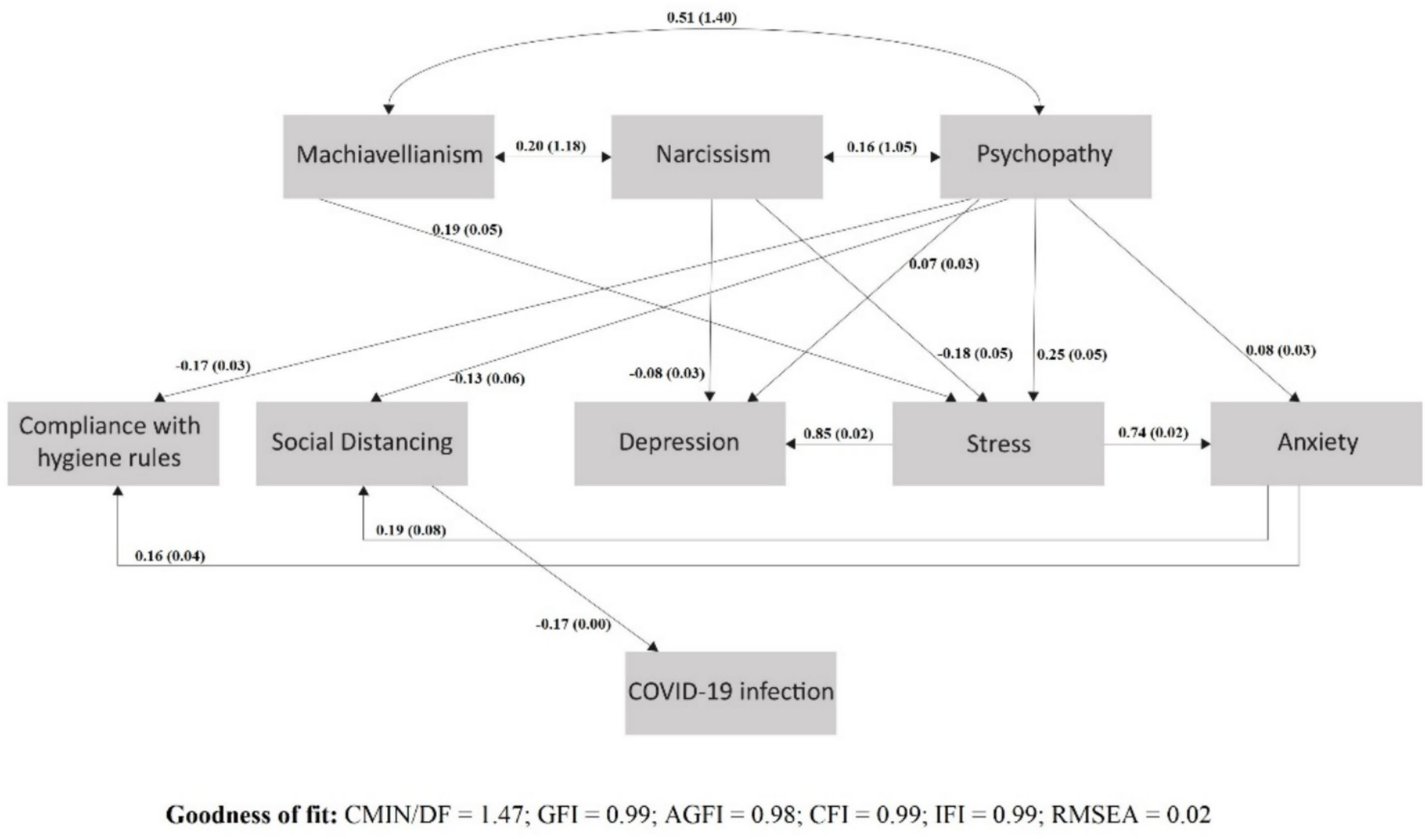
SEM featuring the significant direct and indirect effects of dark personality traits on the COVID-19 infection by mediating social distancing; *β* (S.E).

Figure 3 presents the significant effects of Dark Triad traits on COVID-19 infection via social distancing. We tested all the paths and associations (see Supplementary Table S3). The model had a good fit [Chi-square = 4.42; Degrees of Freedom (DF) = 3; CMIN/DF = 1.47, GFI = 0.99, AGFI = 0.98, CFI = 0.99, IFI = 0.99, RMSEA = 0.02]. SD was negatively associated with COVID-19 infection. Psychopathy had a direct negative association with SD, while other Dark Triad traits were indirectly associated with SD (through stress and anxiety).

In detail, psychopathy was directly and negatively associated with SD (*β* = −0.130; SE = 0.056; *p* = 0.003), and SD was negatively associated with COVID-19 infection (*β* = −0.167; SE = 0.003; *p* < 0.001). Narcissism was not associated directly with SD (*β* = 0.010; SE = 0.053; *p* = 0.795), but it was indirectly linked to it via a negative association with stress (*β* = −0.179; SE = 0.047; *p* < 0.001), and stress was positively linked to anxiety (*β* = 0.738; SE = 0.020; *p* < 0.001). Anxiety in turn had a positive association with SD (*β* = 0.187; SE = 0.077; *p* = 0.001), which was negatively associated with the infection (*β* = −0.167; SE = 0.003; *p* < 0.001). Machiavellianism also was not associated directly with SD (*β* = −0.058; SE = 0.050; *p* = 0.175), but it was indirectly linked to it via a positive association with stress (*β* = 0.192; SE = 0.045; *p* < 0.001), and then followed the same paths of stress, anxiety, SD, and infection. Finally, psychopathy was directly and negatively associated with CHR (*β* = −0.166; SE = 0.029; *p* < 0.001), while Machiavellianism and narcissism were indirectly associated with CHR via stress and anxiety; Machiavellianism had a positive association with stress, while narcissism was negatively associated with stress, which in turn was linked to anxiety (*β* = 0.738; SE = 0.020; *p* < 0.001). Anxiety was positively linked to CHR (*β* = 0.164; SE = 0.040; *p* = 0.005).

## Discussion

We found that SD was a significant negative predictor for COVID-19 infection in our sample, highlighting its key role among all protective behaviors in the dynamics of COVID-19 spread (Kissler et al., 2020). Taking a person-environment transaction perspective (Hopwood et al., 2022), the psychological burden of the COVID-19 pandemic is a new environment in which the individuals, based on their personality traits, may behave differently. Accordingly, in the context of the COVID-19 crisis, we considered the role of individuals’ personality traits in engaging in mitigation behaviors- which is critical to hinder infection. Importantly, the current study was conducted during an accelerating stage of the COVID-19 pandemic and examined mediational models of actual COVID-19 infection. Based on survey data we collected between early November 2021 and late January 2022—a time period that included the strictest government policies in Iran, we found that normal, maladaptive, and dark personality traits followed the same structural paths, in which traits indirectly predicted COVID-19 infection via engagement in, or resistance to, social distancing behaviors. We next consider each of these findings in more detail.

### Normal personality traits

For normative personality traits, we found that openness, conscientiousness, and neuroticism were positively associated with social distancing, whereas extraversion and agreeableness were negatively associated. Subsequently, social distancing as a mediator in turn negatively predicted the COVID-19 infection. Recently, studies from the U.S., U.K., and Germany also examined personality traits as predictors of COVID-19 infection. Peters et al. (2023) indicated that in the early stages of COVID-19 in the US and Germany, the regional level of Openness acted as a risk factor, which is not aligned with our findings. This might be due to the regional level of assessment, different time periods in which data was gathered, or possible cultural differences. They also indicated that the regional level of Neuroticism acted as a protective factor, which is quite aligned with our results. The second study by Rolon et al. (2021) in the US and UK showed that extraversion predisposes people to become infected with the coronavirus, which is also in accordance with our findings.

According to the worldwide scale of the COVID-19 pandemic and the contagiousness of the virus, even small changes in people’s probability of social distancing behaviors such as shelter-in-place can substantially reduce the spread of COVID-19 both within and across countries (Dehning et al., 2020; Kissler et al., 2020). Our findings are wholly in line with conceptual definitions of the Big Five personality traits (DeYoung et al., 2007; Soto and John, 2017) and most prior research. A study conducted in 55 countries revealed that personality independently predicted protective behaviors including sheltering-in-place rates during the COVID-19 pandemic (Gotz et al., 2021). People high in Neuroticism are hyper-vigilant and experience anticipatory anxiety and threat sensitivity (Barlow et al., 2014), consistent with increased social distancing through increased stress and anxiety as we found here.

According to our study, openness positively predicted stress and anxiety, leading to increased social distancing. Although evidence has already shown that openness is related to risky behaviors (Schaller and Murray, 2008), it is also related to accurate risk perceptions- that could be associated with stress and anxiety (Fu and Wang, 2022)- through which openness is linked with increased social distancing behaviors (Trobst et al., 2000). Moreover, we found a direct positive association between conscientiousness and SD. Other studies similarly showed that more conscientious individuals are more likely to follow rules (John and Srivastava, 1999), are more cautious and socially responsible (Roberts et al., 2005), and more likely to consider others’ health (Roberts et al., 2009) which may lead them to comply with strict protective policies (Gotz et al., 2021). In our study, extraversion was a negative predictor of social distancing, which is consistent with its sub-factors of sociability and assertiveness (Soto and John, 2017), and previous research linking this trait to various risky health behaviors (Strickhouser et al., 2017) and decreased germ aversion (Gotz et al., 2021) that is also aligned with our results indicating Extraversion negatively predicted CHR. Further, agreeableness negatively predicted SD via negative association with stress and anxiety. The dimension of agreeableness is clearly represented in conceptions of dependency (Pincus, 2002). Thus, more agreeable people seek more frequent interpersonal contact (McCrae and Costa, 1989; Rollings et al., 2023)- although in contrast to extraverts, they would rather have smaller but more intimate social networks (Harada et al., 2023; Harari et al., 2020)- which can make it harder for them to comply with SD rules (Gotz et al., 2021).

### Maladaptive personality traits

In our study, maladaptive personality traits were negative predictors of social distancing and hygiene rules compliance behaviors, as well as positive predictors of stress, anxiety, and depression. Maladaptive personality traits are positively associated with emotional dysregulation, which in turn is related to symptoms of internalizing disorders such as depression and anxiety (Gratz et al., 2016). In detail, negative affectivity positively predicted SD via increased stress and anxiety, which was consistent with previous studies indicating the association between negative affectivity and SD (Srivastava and Coolidge, 2021) and indicating the relationship between negative affectivity and internalizing psychopathology, such as anxiety (Anderson et al., 2018). Negative affectivity also tends to associate with cold-submissive interpersonal problems, e.g., social avoidance (Wright et al., 2012), which is consistent with increased social distancing in our study.

We found that detachment, in contrast to extraversion positively predicted SD, which is aligned with its features including withdrawal from interpersonal interactions (American Psychiatric Association, 2013), and with prior research during the COVID-19 pandemic (Srivastava and Coolidge, 2021). Consistent with research by Somma et al. (2020) conducted during the COVID-19 pandemic, our findings showed that detachment was associated with higher levels of stress, anxiety, and depression. Antagonism negatively predicted SD and CHR in our sample, which replicates previous findings showing that antagonistic people were less motivated to employ coping strategies, such as SD, during the COVID-19 pandemic (Sica et al., 2021a).

We found that disinhibition, in contrast to conscientiousness, was negatively associated with SD, which is consistent with its features including irresponsibility (American Psychiatric Association, 2013) and prior research showing that disinhibition was negatively related to perceptions of the COVID-19 threat (Sica et al., 2021a), which could lead to decreased motivation to engage in protective behaviors. Prior research also supports our results and indicated a positive direct link between disinhibition and stress (Sica et al., 2021a). Although psychoticism did not directly predict SD, it was linked to SD through psychological responses including stress and anxiety. Psychoticism is associated with feeling disconnected from the real world (Holden et al., 2015) and an increased perception of daily hassles (Compton et al., 2008), which may increase social distancing behaviors.

### Dark triad traits

Prior research found that Dark Triad personality traits were strongly associated with a decreased willingness to comply (Starcevic and Janca, 2022) and non-compliance (Blagov, 2021) with recommendations to limit COVID-19 spread mainly due to resistance to accept personal restrictions. This is aligned with our results in which dark traits negatively predicted SD and CHR. Machiavellianism- which has a strong correlation with low conscientiousness- correlates positively with depression and anxiety symptoms (Jonason et al., 2015), which is consistent with our results. Low sensitivity to threat, affective-interpersonal features of psychopathy, rule-breaking, and disregard for others seems to be associated with decreased engagement in protective and policy-compliant behaviors during the pandemic (Harper et al., 2021; Patrick et al., 2009). Our findings linking psychopathy to anxiety and depression are also consistent with numerous prior investigations (Jonason et al., 2015). We also found that narcissism was negatively associated with stress thereby negatively associated with SD. Similarly, several studies showed that narcissistic people did not experience excessive stress, anxiety, and depression during the COVID-19 pandemic and resisted engaging in protective behaviors (Gogola et al., 2021; Hatemi and Fazekas, 2022), even endorsing conspiracy theories regarding the COVID-19 virus and its vaccines (Hughes and Machan, 2021).

### Cultural considerations

Since personality traits capture individual differences related to social and compliance behaviors through which the virus is transmitted, they can help explain differential transmission of COVID-19, even after controlling for important sociodemographic, economic, and pandemic-related factors (Peters et al., 2020). However, as all countries have suffered the impacts of the COVID-19 pandemic, regional and cultural differences should also be considered. Different countries have diverse cultures that could influence personality and social behaviors, and this could guide government policies encountering public health events, such as COVID-19 pandemic. For example, Gelfand et al. (2021) argued that the US, Canada, and European countries are loose cultures, whereas Asian cultures, as tight cultures, have imposed strict measures and punishments for deviance. Recent research found that compared with nations with high levels of cultural tightness, nations with high levels of cultural looseness had more confirmed COVID-19 cases and deaths (Gelfand et al., 2021). Moreover, cultural collectivism correlates positively with cultural tightness (Gelfand et al., 2011). Recent studies indicated that people from individualistic and collectivistic countries follow governments’ preventive measures (e.g., lockdown, social distancing, using a face mask, etc.) differently to contain the transmission of COVID-19 (Chen and Biswas, 2022; Lee et al., 2021). The studies showed hygiene behaviors such as using masks were higher in more collectivistic countries during the COVID-19 pandemic (Liu et al., 2023). In collectivistic cultures, the importance of “We” surpasses that of “I.” Compared to individual interest, common interest is viewed as ‘in-group,’ rooted in the tightly integrated relationships among families and close friends. As a collectivistic culture, Iranians might adhere to COVID-19 protective behaviors and health guidelines more in the face of the pandemic as a new environment, compared to other countries with individualistic cultures. Consequently, further related studies in different nations, like our study in Iran, can inform public health officials about the important role of individual differences in personality in mitigation behaviors which is critical to hinder infection and also is an influence that is not simply minimized by governmental policy. Public health policymakers can benefit from personality-tailored interventions for maximizing preventive health behaviors (Abdullahi et al., 2020; Boersma et al., 2011; Hagger, 2025; Hirsh et al., 2012; Juchem et al., 2024) and slowing the spread of infection. This knowledge also could contribute to more effective measures to prepare for public health emergencies in the future.

## Limitations, conclusions, and future directions

The results of this study should be considered in light of the following limitations. First, we had to collect the data online due to the regulations enforced by the government during the COVID-19 pandemic. Thus, there was no ability to control the assessment environment or confirm participant responses. Second, our study used self-report assessments because it was not feasible for in-person assessments due to the pandemic-related protective policies. Self-report assessments rely on participants’ self-perceptions, which might have bias. Consequently, the generalizability of the results is potentially limited by the use of the self-report survey methodology. Third, our measure of normal personality traits (BFI-10) is a very brief measure of the Big Five. Finally, self-reported infection was used, thus our infection variable is not laboratory-confirmed. Some possible limits to knowing actual risk of infection include the willingness to be tested and to disclose positive test results. Despite its limitations, the current research suggests that the associations between individual differences in normal, maladaptive, and dark personality traits and COVID-19 infection are mediated through social distancing during COVID-19 pandemic. The results demonstrate the power of personality as a central driver of psychological and behavioral responses to the pandemic (Williams and Carlson, 2025), even in a tight culture such Iran where strict penalties for noncompliance of mitigation regulations are present. Our findings support future research aimed at developing and validating personalized health-related interventions that consider individual differences in personality. Our findings also suggest that interpersonal functioning associated with normal, pathological, and dark personality traits (Ansell and Pincus, 2004; Du et al., 2021; McCrae, 1996; Wright et al., 2012) have a key role in understanding the mediating role of social distancing behaviors between personality and COVID-19 infection. Interpersonal functioning is comprehensively described and explained by the Contemporary Integrative Interpersonal Theory and the interpersonal situation framework (Dawood et al., 2018; Pincus and Hopwood, in press). Future research can use this empirically supported lens to view and examine human functioning in new health-related situations, such as behavioral and psychological responses to acute epidemics and global pandemics.

## Data Availability

The raw data supporting the conclusions of this article will be made available by the authors, without undue reservation.
