# Supplementary material for: Are individual differences in personality associated with COVID-19 infection? Examining the role of normative, maladaptive, and dark personality traits using structural equation modeling
Source: Front Psychol. 2025 Feb 19;16:1511970. doi: 10.3389/fpsyg.2025.1511970 (PMC11879987; doi:10.3389/fpsyg.2025.1511970)
Supplement: Supplementary file 1 [file Table_1.DOCX]

**Are individual differences in personality associated with COVID-19 infection? Examining the role of normative, maladaptive, and dark personality traits using structural equation modeling**

**[Supplemental Materials]**

**Table S1.** Model 1: SEM featuring the direct and indirect effects of normative personality traits on the COVID-19 infection by mediating social distancing.

|  |  |  | **Standardized**  **Coefficients** | **Unstandardized**  **Coefficients** | **S.E** | **C.R** | **P** |
| --- | --- | --- | --- | --- | --- | --- | --- |
| Extraversion | 🡪 | Stress | -.205 | -0.688 | .134 | -5.114 | *** |
| Agreeableness | 🡪 | Stress | -.066 | -0.254 | .124 | -2.046 | .041 |
| Conscientiousness | 🡪 | Stress | -.176 | -0.594 | .111 | -5.365 | *** |
| Neuroticism | 🡪 | Stress | .404 | 1.277 | .104 | 12.324 | *** |
| Openness | 🡪 | Stress | .137 | 0.499 | .145 | 3.435 | *** |
| Extraversion | 🡪 | Depression | -.054 | -0.189 | .080 | -2.367 | .018 |
| Extraversion | 🡪 | Anxiety | -.035 | -0.091 | .080 | -1.132 | .258 |
| Agreeableness | 🡪 | Depression | -.013 | -0.054 | .072 | -.747 | .455 |
| Agreeableness | 🡪 | Anxiety | .016 | 0.047 | .073 | .645 | .519 |
| Conscientiousness | 🡪 | Depression | -.101 | -0.353 | .066 | -5.367 | *** |
| Conscientiousness | 🡪 | Anxiety | .024 | 0.063 | .066 | .943 | .345 |
| Neuroticism | 🡪 | Depression | .007 | 0.022 | .066 | .331 | .741 |
| Neuroticism | 🡪 | Anxiety | .051 | 0.125 | .067 | 1.871 | .061 |
| Openness | 🡪 | Depression | .042 | 0.159 | .085 | 1.865 | .062 |
| Openness | 🡪 | Anxiety | .020 | 0.058 | .086 | .672 | .502 |
| Stress | 🡪 | Anxiety | .754 | 0.590 | .022 | 27.256 | *** |
| Stress | 🡪 | Depression | .828 | 0.859 | .021 | 40.075 | *** |
| Openness | 🡪 | SD | -.004 | -0.016 | .177 | -.088 | .930 |
| Neuroticism | 🡪 | SD | -.021 | -0.070 | .138 | -.511 | .609 |
| Conscientiousness | 🡪 | SD | .144 | 0.502 | .139 | 3.608 | *** |
| Agreeableness | 🡪 | SD | -.007 | -0.030 | .150 | -.200 | .842 |
| Extraversion | 🡪 | SD | -.015 | -0.052 | .166 | -.316 | .752 |
| Extraversion | 🡪 | CHR | -.004 | -0.007 | .086 | -.079 | .937 |
| Agreeableness | 🡪 | CHR | -.014 | -0.028 | .078 | -.364 | .716 |
| Conscientiousness | 🡪 | CHR | .138 | 0.252 | .072 | 3.484 | *** |
| Neuroticism | 🡪 | CHR | .018 | 0.030 | .071 | .426 | .670 |
| Openness | 🡪 | CHR | .012 | 0.024 | .092 | .261 | .794 |
| Stress | 🡪 | CHR | -.102 | -0.055 | .044 | -1.255 | .209 |
| Stress | 🡪 | SD | -.156 | -0.161 | .085 | -1.910 | .056 |
| Anxiety | 🡪 | CHR | .127 | 0.088 | .040 | 2.177 | .029 |
| Anxiety | 🡪 | SD | .146 | 0.193 | .078 | 2.490 | .013 |
| Depression | 🡪 | CHR | -.113 | -0.059 | .041 | -1.445 | .148 |
| Depression | 🡪 | SD | -.011 | -0.011 | .078 | -.137 | .891 |
| Stress | 🡪 | covid19 | .046 | 0.003 | .006 | .571 | .568 |
| Anxiety | 🡪 | covid19 | .069 | 0.006 | .005 | 1.176 | .240 |
| Depression | 🡪 | covid19 | -.005 | 0.000 | .005 | -.059 | .953 |
| CHR | 🡪 | covid19 | .081 | 0.011 | .006 | 1.770 | .077 |
| SD | 🡪 | covid19 | -.167 | -0.012 | .003 | -3.674 | *** |

******* P < 0.001; **P**: P-value; **C.R**: Critical ratio; **S.E**: Standard error; **SD**: Social Distancing; **CHR**: Compliance with hygiene rule.

|  |  |  | **Correlation** | **Covariance** | **S.E** | **C.R** | **P** |
| --- | --- | --- | --- | --- | --- | --- | --- |
| Openness | <--> | Extraversion | 0.625 | 2.446 | .170 | 14.389 | *** |
| Neuroticism | <--> | Extraversion | -0.025 | -0.112 | .166 | -.676 | .499 |
| Conscientiousness | <--> | Extraversion | 0.148 | 0.627 | .157 | 3.990 | *** |
| Agreeableness | <--> | Extraversion | -0.042 | -0.155 | .136 | -1.138 | .255 |
| Openness | <--> | Agreeableness | -0.117 | -0.398 | .126 | -3.156 | .002 |
| Neuroticism | <--> | Agreeableness | -0.213 | -0.836 | .147 | -5.669 | *** |
| Neuroticism | <--> | Conscientiousness | -0.282 | -1.268 | .172 | -7.386 | *** |
| Conscientiousness | <--> | Agreeableness | 0.151 | 0.554 | .137 | 4.057 | *** |
| Openness | <--> | Conscientiousness | 0.049 | 0.193 | .144 | 1.342 | .180 |
| Openness | <--> | Neuroticism | -0.005 | -0.022 | .153 | -.144 | .885 |

*** P < 0.001; **P**: P-value; **C.R**: Critical ratio; **S.E**: Standard error

**Table S2.** Model 2: SEM featuring the direct and indirect effects of maladaptive personality traits on the COVID-19 infection by mediating social distancing.

|  |  |  | **Standardized**  **Coefficients** | **Unstandardized**  **Coefficients** | **S.E** | **C.R** | **P** |
| --- | --- | --- | --- | --- | --- | --- | --- |
| Negative | 🡪 | Stress | .358 | 0.696 | .070 | 9.944 | *** |
| Detachment | 🡪 | Stress | .144 | 0.295 | .067 | 4.374 | *** |
| Antagonism | 🡪 | Stress | .026 | 0.065 | .085 | .757 | .449 |
| Disinhibition | 🡪 | Stress | .091 | 0.190 | .080 | 2.379 | .017 |
| Psychoticism | 🡪 | Stress | .175 | 0.359 | .081 | 4.415 | *** |
| Negative | 🡪 | Depression | .013 | 0.025 | .045 | .568 | .570 |
| Negative | 🡪 | Anxiety | .025 | 0.038 | .046 | .814 | .416 |
| Detachment | 🡪 | Depression | .176 | 0.374 | .041 | 9.112 | *** |
| Detachment | 🡪 | Anxiety | .039 | 0.062 | .042 | 1.474 | .141 |
| Antagonism | 🡪 | Depression | -.021 | -0.055 | .051 | -1.074 | .283 |
| Antagonism | 🡪 | Anxiety | .031 | 0.061 | .053 | 1.142 | .253 |
| Disinhibition | 🡪 | Depression | .004 | 0.008 | .048 | .172 | .863 |
| Disinhibition | 🡪 | Anxiety | .023 | 0.037 | .050 | .737 | .461 |
| Psychoticism | 🡪 | Depression | .050 | 0.105 | .050 | 2.128 | .033 |
| Psychoticism | 🡪 | Anxiety | .115 | 0.185 | .051 | 3.620 | *** |
| Stress | 🡪 | Anxiety | .666 | 0.521 | .023 | 22.809 | *** |
| Stress | 🡪 | Depression | .777 | 0.806 | .022 | 36.431 | *** |
| Stress | 🡪 | SD | -.108 | -0.112 | .085 | -1.314 | .189 |
| Stress | 🡪 | CHR | -.061 | -0.033 | .044 | -.751 | .453 |
| Anxiety | 🡪 | SD | .196 | 0.260 | .078 | 3.329 | *** |
| Anxiety | 🡪 | CHR | .182 | 0.126 | .040 | 3.109 | .002 |
| Depression | 🡪 | SD | -.102 | -0.101 | .081 | -1.259 | .208 |
| Depression | 🡪 | CHR | -.158 | -0.082 | .042 | -1.957 | .050 |
| Negative | 🡪 | CHR | .002 | 0.002 | .050 | .041 | .967 |
| Negative | 🡪 | SD | -.015 | -0.030 | .096 | -.307 | .759 |
| Detachment | 🡪 | CHR | .021 | 0.023 | .048 | .481 | .631 |
| Detachment | 🡪 | SD | .087 | 0.184 | .093 | 1.987 | .047 |
| Antagonism | 🡪 | CHR | -.107 | -0.146 | .056 | -2.587 | .010 |
| Antagonism | 🡪 | SD | -.115 | -0.301 | .110 | -2.731 | .006 |
| Disinhibition | 🡪 | CHR | -.115 | -0.129 | .052 | -2.496 | .013 |
| Disinhibition | 🡪 | SD | -.127 | -0.274 | .103 | -2.671 | .008 |
| Psychoticism | 🡪 | SD | .015 | 0.032 | .088 | .361 | .718 |
| Depression | 🡪 | covid19 | -.005 | 0.000 | .005 | -.059 | .953 |
| Anxiety | 🡪 | covid19 | .069 | 0.006 | .005 | 1.176 | .240 |
| Stress | 🡪 | covid19 | .046 | 0.003 | .006 | .571 | .568 |
| hygiene | 🡪 | covid19 | .081 | 0.011 | .006 | 1.769 | .077 |
| social | 🡪 | covid19 | -.167 | -0.012 | .003 | -3.674 | *** |

*** P < 0.001; **P**: P-value; **C.R**: Critical ratio; **S.E**: Standard error; **SD**: Social Distancing; **CHR**: Compliance with hygiene rule.

|  |  |  | **Correlation** | **Covariance** | **S.E** | **C.R** | **P** |
| --- | --- | --- | --- | --- | --- | --- | --- |
| Psychoticism | <--> | Negative | 0.525 | 6.314 | .500 | 12.640 | *** |
| Disinhibition | <--> | Negative | 0.531 | 6.262 | .491 | 12.742 | *** |
| Antagonism | <--> | Negative | 0.379 | 3.685 | .383 | 9.623 | *** |
| Detachment | <--> | Negative | 0.382 | 4.605 | .475 | 9.692 | *** |
| Psychoticism | <--> | Disinhibition | 0.589 | 6.598 | .478 | 13.805 | *** |
| Antagonism | <--> | Detachment | 0.292 | 2.703 | .355 | 7.609 | *** |
| Disinhibition | <--> | Detachment | 0.379 | 4.263 | .442 | 9.643 | *** |
| Psychoticism | <--> | Detachment | 0.463 | 5.298 | .464 | 11.419 | *** |
| Disinhibition | <--> | Antagonism | 0.457 | 4.146 | .367 | 11.308 | *** |
| Psychoticism | <--> | Antagonism | 0.473 | 4.373 | .376 | 11.633 | *** |

*** P < 0.001; **P**: P-value; **C.R**: Critical ratio; **S.E**: Standard error.

**Table S3.** Model 3: SEM featuring the direct and indirect effects of dark personality traits on the COVID-19 infection by mediating social distancing.

|  |  |  | **Standardized**  **Coefficients** | **Unstandardized**  **Coefficients** | **S.E** | **C.R** | **P** |
| --- | --- | --- | --- | --- | --- | --- | --- |
| Machiavellianism | 🡪 | Stress | .192 | 0.217 | .045 | 4.841 | *** |
| Narcissism | 🡪 | Stress | -.179 | -0.241 | .047 | -5.157 | *** |
| Psychopathy | 🡪 | Stress | .254 | 0.318 | .049 | 6.440 | *** |
| Machiavellianism | 🡪 | Depression | -.033 | -0.038 | .024 | -1.573 | .116 |
| Machiavellianism | 🡪 | Anxiety | .021 | 0.019 | .024 | .777 | .437 |
| Narcissism | 🡪 | Depression | -.079 | -0.111 | .026 | -4.342 | *** |
| Narcissism | 🡪 | Anxiety | -.045 | -0.048 | .025 | -1.865 | .062 |
| Psychopathy | 🡪 | Depression | .068 | -0.089 | .027 | 3.255 | .001 |
| Psychopathy | 🡪 | Anxiety | .075 | 0.074 | .027 | 2.711 | .007 |
| Stress | 🡪 | Anxiety | .738 | 0.577 | .020 | 29.329 | *** |
| Stress | 🡪 | Depression | .854 | 0.887 | .020 | 44.856 | *** |
| Machiavellianism | 🡪 | SD | -.058 | -0.068 | .050 | -1.357 | .175 |
| Machiavellianism | 🡪 | CHR | .035 | 0.022 | .026 | .829 | .407 |
| Narcissism | 🡪 | SD | .010 | 0.014 | .053 | .259 | .795 |
| Narcissism | 🡪 | CHR | .065 | 0.048 | .028 | 1.724 | .085 |
| Psychopathy | 🡪 | SD | -.130 | -0.168 | .056 | -2.986 | .003 |
| Psychopathy | 🡪 | CHR | -.166 | -0.112 | .029 | -3.837 | *** |
| Stress | 🡪 | CHR | -.099 | -0.053 | .043 | -1.226 | .220 |
| Stress | 🡪 | SD | -.135 | -0.139 | .084 | -1.667 | .096 |
| Anxiety | 🡪 | CHR | .164 | 0.113 | .040 | 2.820 | .005 |
| Anxiety | 🡪 | SD | .187 | 0.247 | .077 | 3.195 | .001 |
| Depression | 🡪 | CHR | -.131 | -0.068 | .040 | -1.698 | .089 |
| Depression | 🡪 | SD | -.064 | -0.064 | .077 | -.828 | .408 |
| Depression | 🡪 | covid19 | -.005 | 0.000 | .005 | -.059 | .953 |
| Anxiety | 🡪 | covid19 | .069 | 0.006 | .005 | 1.176 | .240 |
| Stress | 🡪 | covid19 | .046 | 0.003 | .006 | .571 | .568 |
| CHR | 🡪 | covid19 | .081 | 0.011 | .006 | 1.770 | .077 |
| SD | 🡪 | covid19 | -.167 | -0.012 | .003 | -3.674 | *** |

*** P < 0.001; **P**: P-value; **C.R**: Critical ratio; **S.E**: Standard error; **SD**: Social Distancing; **CHR**: Compliance with hygiene rule.

|  |  |  | **Correlation** | **Covariance** | **S.E** | **C.R** | **P** |
| --- | --- | --- | --- | --- | --- | --- | --- |
| Psychopathy | <--> | Machiavellianism | .508 | 17.200 | 1.397 | 12.314 | *** |
| Narcissism | <--> | Machiavellianism | .203 | 6.363 | 1.179 | 5.396 | *** |
| Psychopathy | <--> | Narcissism | .162 | 4.564 | 1.052 | 4.336 | *** |

*** P < 0.001; **P**: P-value; **C.R**: Critical ratio; **S.E**: Standard error.

|  | Extraversion | Agreeableness | Conscientiousness | Neuroticism | Openness | Depression | Anxiety | Stress | SD | CHR |
| --- | --- | --- | --- | --- | --- | --- | --- | --- | --- | --- |
| Extraversion | 1 | -0.042 | .148^**^ | -0.025 | .624^**^ | -.169^**^ | -.136^**^ | -.153^**^ | 0.010 | 0.042 |
| Agreeableness | -0.042 | 1 | .151^**^ | -.213^**^ | -.117^**^ | -.187^**^ | -.133^**^ | -.186^**^ | 0.032 | 0.025 |
| Conscientiousness | .148^**^ | .151^**^ | 1 | -.282^**^ | 0.049 | -.379^**^ | -.236^**^ | -.324^**^ | .166^**^ | .177^**^ |
| Neuroticism | -0.025 | -.213^**^ | -.282^**^ | 1 | -0.005 | .430^**^ | .398^**^ | .472^**^ | -.080^*^ | -0.065 |
| Openness | .624^**^ | -.117^**^ | 0.049 | -0.005 | 1 | 0.010 | 0.002 | 0.006 | -0.006 | 0.017 |
| Depression | -.169^**^ | -.187^**^ | -.379^**^ | .430^**^ | 0.010 | 1 | .745^**^ | .875^**^ | -.098^**^ | -.149^**^ |
| Anxiety | -.136^**^ | -.133^**^ | -.236^**^ | .398^**^ | 0.002 | .745^**^ | 1 | .773^**^ | -0.022 | -0.059 |
| Stress | -.153^**^ | -.186^**^ | -.324^**^ | .472^**^ | 0.006 | .875^**^ | .773^**^ | 1 | -.106^**^ | -.136^**^ |
| SD | 0.010 | 0.032 | .166^**^ | -.080^*^ | -0.006 | -.098^**^ | -0.022 | -.106^**^ | 1 | .602^**^ |
| CHR | 0.042 | 0.025 | .177^**^ | -0.065 | 0.017 | -.149^**^ | -0.059 | -.136^**^ | .602^**^ | 1 |

**Table S4.** Bivariate Pearson correlation among variables in model 1.

****** P < 0.01; *****P < 0.05; **SD**: Social Distancing; **CHR**: Compliance with hygiene rule.

**Table S5.** Bivariate Pearson correlation among variables in model 2.

|  | Negative Affectivity | Detachment | Antagonism | Disinhibition | Psychoticism | Depression | Anxiety | Stress | SD | CHR |
| --- | --- | --- | --- | --- | --- | --- | --- | --- | --- | --- |
| Negative Affectivity | 1 | .382** | .379** | .531** | .525** | .537** | .499** | .563** | -.102** | -.120** |
| Detachment | .382** | 1 | .292** | .379** | .463** | .513** | .388** | .404** | -0.013 | -.088* |
| Antagonism | .379** | .292** | 1 | .457** | .473** | .315** | .334** | .328** | -.148** | -.162** |
| Disinhibition | .531** | .379** | .457** | 1 | .589** | .447** | .433** | .451** | -.155** | -.174** |
| Psychoticism | .525** | .463** | .473** | .589** | 1 | .515** | .504** | .496** | -.117** | -.176** |
| Depression | .537** | .513** | .315** | .447** | .515** | 1 | .745** | .875** | -.098** | -.149** |
| Anxiety | .499** | .388** | .334** | .433** | .504** | .745** | 1 | .773** | -0.022 | -0.059 |
| Stress | .563** | .404** | .328** | .451** | .496** | .875** | .773** | 1 | -.106** | -.136** |
| SD | -.102** | -0.013 | -.148** | -.155** | -.117** | -.098** | -0.022 | -.106** | 1 | .602** |
| CHR | -.120** | -.088* | -.162** | -.174** | -.176** | -.149** | -0.059 | -.136** | .602** | 1 |

****** P < 0.01; *****P < 0.05; **SD**: Social Distancing; **CHR**: Compliance with hygiene rule.

**Table S6.** Bivariate Pearson correlation among variables in model 3.

|  | Machiavellianism | Narcissism | Psychopathy | Depression | Anxiety | Stress | SD | CHR |
| --- | --- | --- | --- | --- | --- | --- | --- | --- |
| Machiavellianism | 1 | .203** | .508** | .229** | .261** | .285** | -.127** | -0.051 |
| Narcissism | .203** | 1 | .162** | -.159** | -.101** | -.099** | -0.018 | 0.059 |
| Psychopathy | .508** | .162** | 1 | .314** | .317** | .322** | -.162** | -.158** |
| Depression | .229** | -.159** | .314** | 1 | .745** | .875** | -.098** | -.149** |
| Anxiety | .261** | -.101** | .317** | .745** | 1 | .773** | -0.022 | -0.059 |
| Stress | .285** | -.099** | .322** | .875** | .773** | 1 | -.106** | -.136** |
| SD | -.127** | -0.018 | -.162** | -.098** | -0.022 | -.106** | 1 | .602** |
| CHR | -0.051 | 0.059 | -.158** | -.149** | -0.059 | -.136** | .602** | 1 |

****** P < 0.01; **SD**: Social Distancing; **CHR**: Compliance with hygiene rule.

**Table S7.** Independent-samples t-test based on sex

|  | Sex | Mean | S.D | Sig. (2-tailed) | Mean Difference | 95% CI | |
| --- | --- | --- | --- | --- | --- | --- | --- |
|  |  |  |  |  |  | Lower | Upper |
| Extraversion | Female | 6.1745 | 2.11545 | 0.386 | 0.14067 | -0.17803 | 0.45936 |
|  | Male | 6.0338 | 1.92425 |  |  |  |  |
| Agreeableness | Female | 6.3846 | 1.76971 | 0.000 | -0.75065 | -1.03377 | -0.46754 |
|  | Male | 7.1353 | 1.73794 |  |  |  |  |
| Conscientiousness | Female | 6.3621 | 2.03776 | 0.000 | -1.12582 | -1.44579 | -0.80586 |
|  | Male | 7.4879 | 1.86136 |  |  |  |  |
| Neuroticism | Female | 7.0713 | 2.11923 | 0.000 | 1.57371 | 1.24002 | 1.90740 |
|  | Male | 5.4976 | 1.95799 |  |  |  |  |
| Openness | Female | 7.4878 | 1.88642 | 0.292 | 0.16413 | -0.14171 | 0.46997 |
|  | Male | 7.3237 | 1.94252 |  |  |  |  |
| Negative Affectivity | Female | 8.5478 | 3.46031 | 0.000 | 2.07441 | 1.52119 | 2.62763 |
|  | Male | 6.4734 | 3.39017 |  |  |  |  |
| Detachment | Female | 5.5760 | 3.42321 | 0.001 | 0.91898 | 0.37730 | 1.46066 |
|  | Male | 4.6570 | 3.22509 |  |  |  |  |
| Antagonism | Female | 4.3471 | 2.77570 | 0.027 | 0.49685 | 0.05805 | 0.93565 |
|  | Male | 3.8502 | 2.60541 |  |  |  |  |
| Disinhibition | Female | 5.4428 | 3.36376 | 0.000 | 1.03215 | 0.52329 | 1.54101 |
|  | Male | 4.4106 | 3.07834 |  |  |  |  |
| Psychoticism | Female | 5.5422 | 3.45059 | 0.011 | 0.67748 | 0.15705 | 1.19791 |
|  | Male | 4.8647 | 3.14397 |  |  |  |  |
| Machiavellianism | Female | 25.5722 | 6.18762 | 0.126 | 0.77030 | -0.21601 | 1.75661 |
|  | Male | 24.8019 | 5.99550 |  |  |  |  |
| Narcissism | Female | 26.9287 | 5.26181 | 0.689 | -0.16791 | -0.99210 | 0.65628 |
|  | Male | 27.0966 | 4.75827 |  |  |  |  |
| Psychopathy | Female | 20.6435 | 5.55429 | 0.015 | -1.10043 | -1.98502 | -0.21585 |
|  | Male | 21.7440 | 5.36413 |  |  |  |  |
| Depression | Female | 10.3415 | 7.12665 | 0.000 | 4.19170 | 3.12292 | 5.26049 |
|  | Male | 6.1498 | 6.43955 |  |  |  |  |
| Anxiety | Female | 6.4315 | 5.55843 | 0.000 | 3.14650 | 2.39498 | 3.89801 |
|  | Male | 3.2850 | 4.27580 |  |  |  |  |
| Stress | Female | 11.3021 | 6.70601 | 0.000 | 4.74168 | 3.70949 | 5.77387 |
|  | Male | 6.5604 | 6.29262 |  |  |  |  |
| Protective Behaviors | Female | 35.8086 | 9.42825 | 0.049 | 1.69752 | 0.00708 | 3.38796 |
|  | Male | 34.1111 | 10.87851 |  |  |  |  |
| Social Distancing | Female | 19.8593 | 7.03889 | 0.037 | 1.22161 | 0.07213 | 2.37108 |
|  | Male | 18.6377 | 7.42726 |  |  |  |  |
| CHR | Female | 15.9493 | 3.52692 | 0.151 | 0.47591 | -0.17491 | 1.12674 |
|  | Male | 15.4734 | 4.22177 |  |  |  |  |

**S.D**: Standard Deviation; **CI**: Confidence Interval of the Difference; **CHR**: Compliance with hygiene rule.

**Table S8.** Independent-samples t-test based on History of Mental Disorder

|  | History of mental health | Mean | S.D | Sig. (2-tailed) | Mean Difference | 95% CI | |
| --- | --- | --- | --- | --- | --- | --- | --- |
|  |  |  |  |  |  | Lower | Upper |
| Extraversion | NO | 6.1122 | 2.04239 | 0.553 | -0.11144 | -0.48020 | 0.25732 |
|  | YES | 6.2237 | 2.14753 |  |  |  |  |
| Agreeableness | NO | 6.7143 | 1.75889 | 0.000 | 0.58271 | 0.26521 | 0.90021 |
|  | YES | 6.1316 | 1.84744 |  |  |  |  |
| Conscientiousness | No | 6.8895 | 1.98453 | 0.000 | 1.03419 | 0.67507 | 1.39331 |
|  | Yes | 5.8553 | 2.10772 |  |  |  |  |
| Neuroticism | No | 6.4405 | 2.17971 | 0.000 | -0.92794 | -1.31386 | -0.54203 |
|  | Yes | 7.3684 | 2.08331 |  |  |  |  |
| Openness | No | 7.3878 | 1.91541 | 0.128 | -0.26356 | -0.60309 | 0.07597 |
|  | Yes | 7.6513 | 1.84233 |  |  |  |  |
| Negative Affectivity | No | 7.6786 | 3.55206 | 0.000 | -1.40695 | -2.03561 | -0.77830 |
|  | Yes | 9.0855 | 3.38861 |  |  |  |  |
| Detachment | No | 4.9235 | 3.20178 | 0.000 | -1.92521 | -2.56759 | -1.28284 |
|  | Yes | 6.8487 | 3.67335 |  |  |  |  |
| Antagonism | No | 4.0595 | 2.68345 | 0.004 | -0.72337 | -1.20974 | -0.23701 |
|  | Yes | 4.7829 | 2.87007 |  |  |  |  |
| Disinhibition | No | 4.8639 | 3.28517 | 0.000 | -1.41237 | -1.99644 | -0.82830 |
|  | Yes | 6.2763 | 3.20843 |  |  |  |  |
| Psychoticism | No | 5.0238 | 3.33728 | 0.000 | -1.60119 | -2.19407 | -1.00831 |
|  | Yes | 6.6250 | 3.24662 |  |  |  |  |
| Machiavellianism | No | 25.1871 | 6.06964 | 0.139 | -0.82608 | -1.92206 | 0.26989 |
|  | Yes | 26.0132 | 6.38395 |  |  |  |  |
| Narcissism | No | 27.1190 | 4.78955 | 0.200 | 0.69799 | -0.37285 | 1.76884 |
|  | Yes | 26.4211 | 6.23654 |  |  |  |  |
| Psychopathy | No | 20.4898 | 5.30965 | 0.000 | -2.24705 | -3.22038 | -1.27371 |
|  | Yes | 22.7368 | 5.95873 |  |  |  |  |
| Depression | No | 8.3078 | 7.06929 | 0.000 | -4.19218 | -5.44084 | -2.94351 |
|  | Yes | 12.5000 | 6.67307 |  |  |  |  |
| Anxiety | No | 4.9541 | 5.23156 | 0.000 | -2.90776 | -3.85287 | -1.96265 |
|  | Yes | 7.8618 | 5.51464 |  |  |  |  |
| Stress | No | 9.1514 | 6.86937 | 0.000 | -4.01311 | -5.14861 | -2.87762 |
|  | Yes | 13.1645 | 6.19169 |  |  |  |  |
| Protective Behaviors | No | 35.6990 | 9.72936 | 0.048 | 1.77793 | 0.01697 | 3.53888 |
|  | Yes | 33.9211 | 10.34218 |  |  |  |  |
| Social Distancing | No | 19.7415 | 7.04775 | 0.095 | 1.09018 | -0.18829 | 2.36865 |
|  | Yes | 18.6513 | 7.56627 |  |  |  |  |
| CHR | No | 15.9575 | 3.69307 | 0.043 | 0.68775 | 0.02150 | 1.35399 |
|  | Yes | 15.2697 | 3.86866 |  |  |  |  |

**S.D**: Standard Deviation; **CI**: Confidence Interval of the Difference; **CHR**: Compliance with hygiene rule.

**Table S9.** Independent-samples t-test based on History of medical disease

|  | History of medical disease | Mean | S.D | Sig. (2-tailed) | Mean Difference | 95% CI | |
| --- | --- | --- | --- | --- | --- | --- | --- |
|  |  |  |  |  |  | Lower | Upper |
| Extraversion | NO | 6.1252 | 2.02726 | 0.846 | -0.03196 | -0.35431 | 0.29039 |
|  | YES | 6.1572 | 2.14634 |  |  |  |  |
| Agreeableness | NO | 6.6243 | 1.79316 | 0.501 | 0.09588 | -0.18394 | 0.37570 |
|  | YES | 6.5284 | 1.79065 |  |  |  |  |
| Conscientiousness | No | 6.7436 | 1.98594 | 0.187 | 0.21526 | -0.10494 | 0.53545 |
|  | Yes | 6.5284 | 2.18955 |  |  |  |  |
| Neuroticism | No | 6.5421 | 2.19842 | 0.099 | -0.28762 | -0.62931 | 0.05407 |
|  | Yes | 6.8297 | 2.16669 |  |  |  |  |
| Openness | No | 7.4990 | 1.87436 | 0.223 | 0.18461 | -0.11228 | 0.48151 |
|  | Yes | 7.3144 | 1.96165 |  |  |  |  |
| Negative Affectivity | No | 7.8826 | 3.59328 | 0.333 | -0.27462 | -0.83082 | 0.28157 |
|  | Yes | 8.1572 | 3.49331 |  |  |  |  |
| Detachment | No | 5.2387 | 3.24275 | 0.362 | -0.25907 | -0.81693 | 0.29879 |
|  | Yes | 5.4978 | 3.70470 |  |  |  |  |
| Antagonism | No | 4.1898 | 2.70243 | 0.786 | -0.05908 | -0.48657 | 0.36840 |
|  | Yes | 4.2489 | 2.81663 |  |  |  |  |
| Disinhibition | No | 5.0705 | 3.27901 | 0.306 | -0.27016 | -0.78797 | 0.24765 |
|  | Yes | 5.3406 | 3.39984 |  |  |  |  |
| Psychoticism | No | 5.2877 | 3.35153 | 0.435 | -0.21015 | -0.73784 | 0.31755 |
|  | Yes | 5.4978 | 3.44331 |  |  |  |  |
| Machiavellianism | No | 25.4247 | 6.09964 | 0.653 | 0.21942 | -0.73969 | 1.17852 |
|  | Yes | 25.2052 | 6.24056 |  |  |  |  |
| Narcissism | No | 27.1507 | 5.01398 | 0.165 | 0.56553 | -0.23380 | 1.36486 |
|  | Yes | 26.5852 | 5.34986 |  |  |  |  |
| Psychopathy | No | 20.8317 | 5.47786 | 0.379 | -0.38664 | -1.24858 | 0.47531 |
|  | Yes | 21.2183 | 5.61681 |  |  |  |  |
| Depression | No | 8.9687 | 7.19088 | 0.258 | -0.64703 | -1.76898 | 0.47492 |
|  | Yes | 9.6157 | 7.17702 |  |  |  |  |
| Anxiety | No | 5.3757 | 5.37895 | 0.188 | -0.56750 | -1.41264 | 0.27764 |
|  | Yes | 5.9432 | 5.49013 |  |  |  |  |
| Stress | No | 9.8317 | 6.85044 | 0.399 | -0.46524 | -1.54647 | 0.61599 |
|  | Yes | 10.2969 | 7.09145 |  |  |  |  |
| Protective Behaviors | No | 35.1820 | 9.75123 | 0.533 | -0.49049 | -2.03315 | 1.05216 |
|  | Yes | 35.6725 | 10.16669 |  |  |  |  |
| Social Distancing | No | 19.3620 | 7.09294 | 0.378 | -0.50259 | -1.62143 | 0.61625 |
|  | Yes | 19.8646 | 7.32899 |  |  |  |  |
| CHR | No | 15.8200 | 3.69457 | 0.968 | 0.01210 | -0.57178 | 0.59598 |
|  | Yes | 15.8079 | 3.83974 |  |  |  |  |

**S.D**: Standard Deviation; **CI**: Confidence Interval of the Difference; **CHR**: Compliance with hygiene rule.

**Table S10.** Independent-samples t-test based on COVID-19 vaccination

|  | COVID-19 Vaccination | Mean | S.D | Sig. (2-tailed) | Mean Difference | **95% CI** | |
| --- | --- | --- | --- | --- | --- | --- | --- |
|  |  |  |  |  |  | Lower | Upper |
| Extraversion | NO | 6.0636 | 2.04628 | 0.377 | -0.13439 | -0.43289 | 0.16412 |
|  | YES | 6.1980 | 2.07895 |  |  |  |  |
| Agreeableness | NO | 6.6474 | 1.86111 | 0.453 | 0.09918 | -0.16006 | 0.35841 |
|  | YES | 6.5482 | 1.72954 |  |  |  |  |
| Conscientiousness | No | 6.7630 | 2.06599 | 0.286 | 0.16148 | -0.13530 | 0.45826 |
|  | Yes | 6.6015 | 2.03933 |  |  |  |  |
| Neuroticism | No | 6.5520 | 2.14379 | 0.358 | -0.14848 | -0.46546 | 0.16849 |
|  | Yes | 6.7005 | 2.23246 |  |  |  |  |
| Openness | No | 7.3728 | 1.93586 | 0.355 | -0.12971 | -0.40490 | 0.14548 |
|  | Yes | 7.5025 | 1.87287 |  |  |  |  |
| Negative Affectivity | No | 7.7717 | 3.57567 | 0.161 | -0.36792 | -0.88287 | 0.14704 |
|  | Yes | 8.1396 | 3.54661 |  |  |  |  |
| Detachment | No | 5.3699 | 3.27071 | 0.702 | 0.09583 | -0.39508 | 0.58674 |
|  | Yes | 5.2741 | 3.49860 |  |  |  |  |
| Antagonism | No | 4.0578 | 2.68805 | 0.162 | -0.28230 | -0.67785 | 0.11325 |
|  | Yes | 4.3401 | 2.77504 |  |  |  |  |
| Disinhibition | No | 4.9942 | 3.41777 | 0.220 | -0.30020 | -0.77979 | 0.17940 |
|  | Yes | 5.2944 | 3.22358 |  |  |  |  |
| Psychoticism | No | 5.2254 | 3.37012 | 0.337 | -0.23903 | -0.72784 | 0.24977 |
|  | Yes | 5.4645 | 3.38760 |  |  |  |  |
| Machiavellianism | No | 25.0116 | 6.06533 | 0.152 | -0.64834 | -1.53583 | 0.23915 |
|  | Yes | 25.6599 | 6.19703 |  |  |  |  |
| Narcissism | No | 27.1185 | 5.08426 | 0.478 | 0.26824 | -0.47305 | 1.00953 |
|  | Yes | 26.8503 | 5.16057 |  |  |  |  |
| Psychopathy | No | 21.0058 | 5.58310 | 0.802 | 0.10223 | -0.69674 | 0.90120 |
|  | Yes | 20.9036 | 5.47126 |  |  |  |  |
| Depression | No | 8.4653 | 7.21112 | 0.012 | -1.32148 | -2.35747 | -0.28550 |
|  | Yes | 9.7868 | 7.11950 |  |  |  |  |
| Anxiety | No | 4.9711 | 5.29909 | 0.006 | -1.08982 | -1.86979 | -0.30984 |
|  | Yes | 6.0609 | 5.47317 |  |  |  |  |
| Stress | No | 9.2197 | 6.89198 | 0.005 | -1.41994 | -2.41691 | -0.42297 |
|  | Yes | 10.6396 | 6.89338 |  |  |  |  |
| Protective Behaviors | No | 36.4017 | 10.12776 | 0.006 | 2.00580 | 0.58353 | 3.42806 |
|  | Yes | 34.3959 | 9.56699 |  |  |  |  |
| Social Distancing | No | 20.4682 | 7.18496 | 0.001 | 1.78547 | 0.75638 | 2.81455 |
|  | Yes | 18.6827 | 7.05257 |  |  |  |  |
| CHR | No | 15.9335 | 4.00126 | 0.428 | 0.22033 | -0.32524 | 0.76590 |
|  | Yes | 15.7132 | 3.49139 |  |  |  |  |

**S.D**: Standard Deviation; **CI**: Confidence Interval of the Difference; **CHR**: Compliance with hygiene rule.
